# Robust and cost-effective silver dendritic nanostructures for SERS-based trace detection of RDX and ammonium nitrate

**DOI:** 10.1039/d0ra08834j

**Published:** 2020-12-17

**Authors:** V. S. Vendamani, S. V. S. Nageswara Rao, A. P. Pathak, Venugopal Rao Soma

**Affiliations:** Advanced Centre for Research in High Energy Materials (ACRHEM), University of Hyderabad Hyderabad 500046 India soma_venu@uohyd.ac.in somavenu@gmail.com; Centre for Advanced Studies in Electronics Science and Technology (CASEST), University of Hyderabad Hyderabad 500046 Telangana India; School of Physics, University of Hyderabad Hyderabad 500046 Telangana India

## Abstract

We report the fabrication and performance evaluation of cost-effective, reproducible silver nanodendrite (AgND) substrates, possessing high-density trunks and branches, achieved by a simple electroless etching process and subsequently utilized them for the trace detection of 1,3,5-trinitroperhydro-1,3,5-triazine (Research Development Explosive, RDX) and Ammonium Nitrate (AN). The intricate structural features in AgNDs offer high-density hotspots for effective molecular detection based on the surface enhanced Raman scattering (SERS) technique. The active SERS-substrate was initially tested with standard Rhodamine 6G (R6G) molecules at 1 nM concentration, which established an effective enhancement factor (EF) of ∼10^8^. The AgNDs were subsequently utilized in the detection of the explosives RDX and AN, down to concentrations of 1 μM. The typical EF achieved in the case of RDX and AN was ∼10^4^. The sensitivity of 1 μM R6G was further enhanced by two-fold through the deposition of Au nanoparticles on the AgNDs. The reproducibility of the low-cost substrate was also demonstrated, with a ∼9% RSD value in the measurements.

## Abstract 2

We report the fabrication and performance evaluation of cost-effective, reproducible silver nanodendrite (AgND) substrates, possessing high-density trunks and branches, achieved by a simple electroless etching and used for the trace detection of RDX and Ammonium Nitrate.

## Graphical abstract

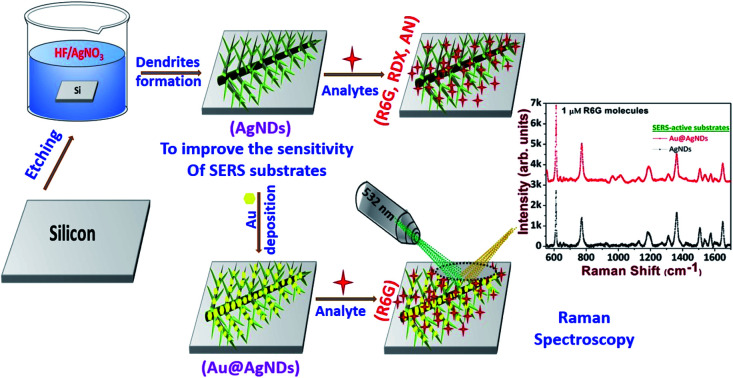

## 1. Introduction

Raman spectroscopy has proven to be a versatile analytical tool in recent years, with several practical applications. This technique has the capability to identify chemical and biological molecules in many areas such as chemical production, biochemistry, food safety, and environmental pollution.^1–6^ However, it has limitations in terms of the sensitivity of molecular detection at trace levels, due to its limited scattering cross-section. This phenomenon has been extensively applied to metal nanostructures for effective molecular detection *viz.* the localized surface plasmon resonance effect. This is termed surface-enhanced Raman spectroscopy (SERS). SERS has been proven to have a tremendous impact on the detection capability, even down to the single-molecule level, with enhancements as large as 10^14^.^7–9^ The SERS enhancement is fundamentally correlated with two kinds of mechanisms: chemical (CM) and electromagnetic (EM) enhancements.^10–12^ In the present scenario, a challenging task is to fabricate affordable, stable, and reproducible SERS-active substrates with a sufficient number of hotspots to achieve sensitive detection. Therefore, the fabrication of ordered and self-aligned metal nanostructures (NSs) is essential to enrich the detection capabilities. In this context, among the available metal NSs, silver (Ag) has demonstrated outstanding advancements in diverse fields such as sensing, catalysis, and optoelectronics, *etc.*,^13–19^ due to its effective enhancement factor, and ability for single-molecule detection.^20^

Recently, many researchers have made progressive accomplishments in the fabrication of metal AgNSs with complex morphologies, *viz.* lithography, electro-deposition, laser ablation, focused ion beams, and wet chemistry, *etc.*^21–25^ Among the various AgNSs, silver nanodendrites (AgNDs) have a pronounced structure that offers many advantages, as their abundant trunks and branches provide scope for a large number of hotspots, which can boost the detection capability and sensitivity.^26–29^ Ag dendrites can be prepared by an electroless deposition process (or galvanic displacement), which is a streamlined wetting process that utilizes a solid reducing agent, such as a silicon wafer, copper plate, aluminium foil, and zinc templates, to name a few.^30–33^ A silicon wafer can be utilized as a reducing agent due to its compatibility, and well-understood chemical and physical properties.^34^ This process is often used for making highly-branched AgNDs, due to its cost-effectiveness, simple methodology, and quality of production. The fascinating optical features of Ag nanoparticles (NPs) and NSs are deeply related to their size, shape, dimension, and inter-particle distance.^35,36^ In future, it is imperative to achieve the ability to tune the morphology of NDs in a controlled manner. Furthermore, thermodynamic factors involved in the etching process also account for fractal changes observed in AgNDs.^37^ In this article, we have investigated the effect of electrolyte concentration and temperature on the modulation of AgND morphology. In further detailed studies, we explored the optimization of AgNDs for sensing applications. These SERS-active substrates were tested initially with Rhodamine 6G (R6G) molecules. Subsequently, we tested the efficacy of these substrates with two well-known explosive molecules, 1,3,5-trinitroperhydro-1,3,5-triazine (Research Development Explosive, RDX) and Ammonium Nitrate (AN). A comparison of the performance of our substrates with some recently reported SERS substrates is also presented.

## 2. Experimental details

Silver nanodendrites were synthesized on a silicon wafer as a solid reducing agent in electroless or galvanic deposition, which is one of the prominent and user-friendly methods to integrate highly branched AgNDs in realistic time. The p-type, boron-doped, single-crystal, 1–10 Ω cm, commercially obtained Si wafers were initially cleaned with acetone to remove the organic residues. Si samples were subsequently dipped into diluted 10% HF solution to etch native oxide. The properly scrubbed Si samples were subjected to electroless deposition. In this process, the density of branches and trunks can be controlled by tuning the etching parameters, such as AgNO_3_ concentration in the electrolyte, etching time, and deposition temperature.^37–40^ In this principle, the surface coverage of AgNPs on Si was investigated by varying the AgNO_3_ molar concentration from 5 mM (0.8 μg μL^−1^) to 25 mM (4.2 μg μL^−1^) in a 5 mM interval range in conjunction with 5 mM HF for 15 minutes of etching time at room temperature. The optimized AgNO_3_ concentration was explored to investigate the kinetics of AgND growth at various temperatures between 25 °C and 60 °C. At the optimized AgNO_3_ concentration and etching temperature, a 3-inch Si wafer costing 25 US$ was processed to make a SERS substrate with well-dispersed AgNDs. This substrate was further fragmented into 1 × 1 cm^2^ pieces for scalable investigations. The resulting ∼1 cm^2^ substrate costs less than 5 US$, and we believe it is a robust, low-cost SERS-active substrate for trace-level detection of hazardous materials. Further, the stability and oxidation rate of the AgND substrate can be improved by gold nanoparticle (AuNPs) decoration. An optimized mixture of 10 mM (3.3 μg μL^−1^) HAuCl_4_ : 3H_2_O with 5 mM HF was used for uniform Au deposition on the AgND SERS substrates. The morphology of the Au-coated AgNDs (Au@AgNDs) was investigated using a conventional field emission scanning electron microscope (FESEM). The electron diffraction data along with FESEM data confirmed the presence of active elements (Ag, Au, Si, and O). The successfully fabricated high-quality SERS-active AgNDs were primarily tested using the standard dye molecule R6G in concentrations of 50 μM to 1 nM. Furthermore, the capabilities and sensitivity of these substrates were tested using the explosives RDX and AN. To perform SERS measurements, 20 μL of the analyte was dripped onto AgNDs with a spreading area of 1 × 1 cm^2^. The samples were dried at room temperature. The Raman spectra were recorded with 532 nm laser excitation.

## 3. Results and discussion

**Fig. 1:**
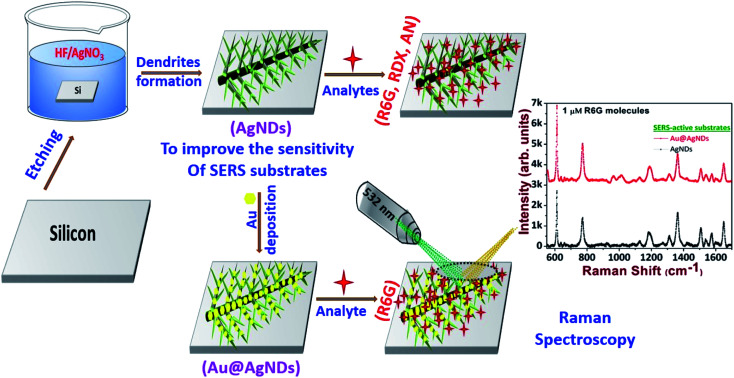
Schematic representation of the experimental process to achieve AgNDs and Au@AgNDs.

**Fig. 2:**
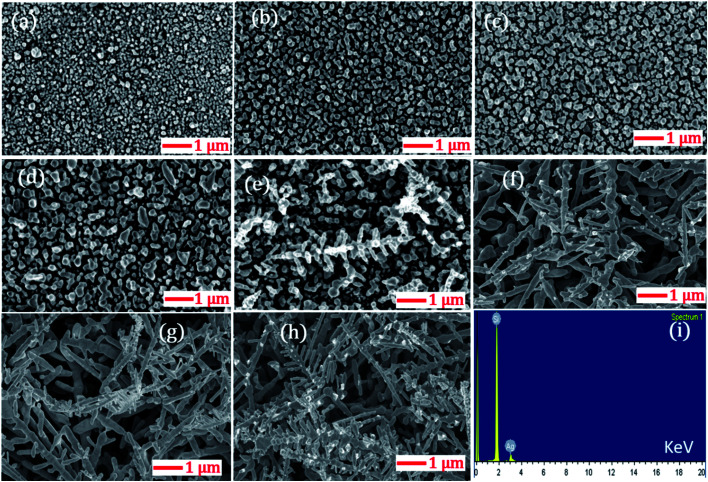
Morphological evolution of AgNDs at various AgNO_3_ concentrations: (a) 5 mM (0.8 μg μL^−1^); (b) 10 mM (1.6 μg μL^−1^); (c) 15 mM (2.5 μg μL^−1^); (d) 20 mM (3.3 μg μL^−1^); (e) 25 mM (4.2 μg μL^−1^); (f) 30 mM (5.09 μg μL^−1^); (g) 35 mM (5.94 μg μL^−1^); (h) 40 mM (6.79 μg μL^−1^). (i) Corresponding EDS spectrum.

The highly branched plasmonic silver nanodendrites were fabricated by electroless deposition for efficient molecular detection. The schematic representation of the experimental procedure is illustrated in Fig. 1. The FESEM image shown in Fig. 2 presents the remarkable plasmonic silver nanodendrites with extensive symmetrical branches and trunks, prepared at various AgNO_3_ concentrations and at room temperature. As shown in Fig. 2a and b, the formation of AgNPs was observed at lower concentrations of AgNO_3_. As the concentration of AgNO_3_ increases, the inter-particle separation decreases (Fig. 2c and d), and nearby particle binding commences. The AgNO_3_ concentration determines the surface coverage of AgNPs on the Si wafer.

Upon utilizing higher AgNO_3_ concentrations of 25 mM, 30 mM, 35 mM and 40 mM (data shown in Fig. 2e–h), the seamless morphological transformation from nanoparticles to nanodendrites was realized. Slightly disordered structures exhibiting inhomogeneity of the AgNDs and a lower concentration of nanoparticles were observed at higher concentrations based on the diffusion-limited aggregation model.^41^ According to this model, the backbone of the dendrites formed is due to continuous diffusion towards the anisotropic aggregation of free Ag nanoparticles. As a continuous reaction, the growth preferentially occurs at the tips and stems of the branches. Furthermore, we also observed truncated dendrites at higher concentrations. After evaluating all the experimental parameters and cost-effectiveness, we realized that 25 mM AgNO_3_ is an optimized concentration for AgND formation. The nanodendrites prepared at 25 mM were highly symmetrical with an estimated length of 1.2–3 μm. Following these measurements, we confirmed that 25 mM was indeed the appropriate concentration for achieving dendrites with long branches and an optimum number of hotspots. The angle between the stem and branches varied in the 52–65° range. Energy dispersive X-ray spectroscopy (EDS) was performed to examine the elemental compositions of AgNDs, which resulted in silicon and silver, as shown in Fig. 2i.

**Fig. 3:**
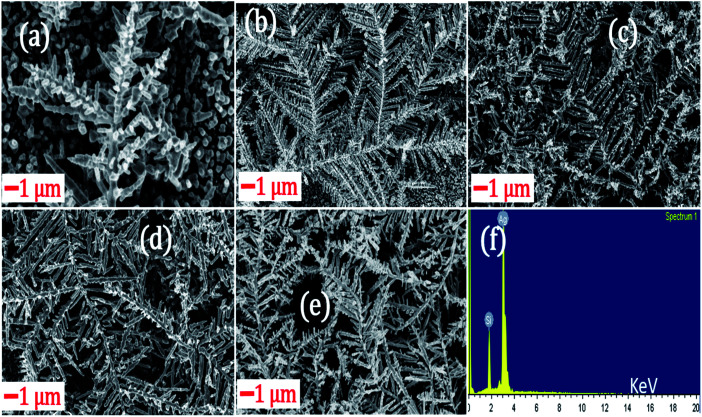
The modulation of AgNDs at various AgNO_3_ deposition temperatures: (a) 25 °C; (b) 30 °C; (c) 40 °C; (d) 50 °C; (e) 60 °C. (f) Corresponding elemental confirmation through EDX data.

This investigation was extended to explore the temperature dependence on the modulation of dendritic nanostructures. Fig. 3 shows the morphological changes as a function of silver deposition temperature in the range of 25 °C to 60 °C. Initially, silver deposition was performed at 25 °C and the budding of AgNDs formation was realized. By increasing the silver deposition temperature to 30 °C, a complex dendritic structure formation with trunks, branches, and sub-branches in controlled growth was observed. When the temperature was raised to 40 °C, the density of sub-branches was found to be reduced when compared to lower temperature.

**Fig. 4:**
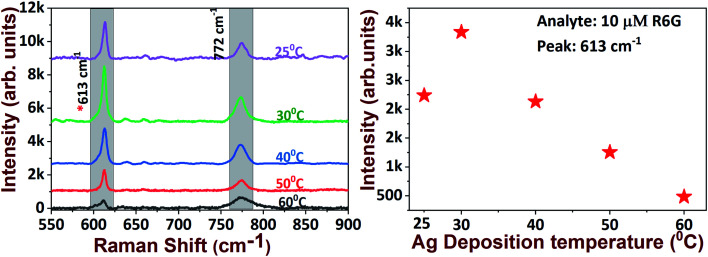
R6G molecular detection on various SERS substrates grown at different deposition temperatures (left) and the intensity of the 613 cm^−1^ peak with increasing deposition temperature (right).

Increasing the temperature to 50 °C did not lead to any significant morphological changes compared with the 40 °C deposition temperature. In addition, the destruction of the formation of silver dendritic branches was observed as the temperature was further increased to 60 °C. The observed changes in the morphological characteristics of AgNDs upon varying the deposition temperatures might be due to the production and or of holes by the reducing agent during the etching process. We found that well-ordered AgNDs were formed at the threshold temperature at 30 °C on the basis of primary investigations with R6G molecular detection. We assessed the capabilities of AgNDs prepared at various deposition temperatures for the detection of 10 μM R6G. The observed Raman data suggest that the intensity of the 613 cm^−1^ Raman mode decreased with increasing deposition temperature (as illustrated in Fig. 4). This observation could possibly be due to the reduced number of electric field efficient spots (*i.e.* a reduced density of sub-branches) in the sample, which are the factors responsible for Raman enhancements.

**Fig. 5:**
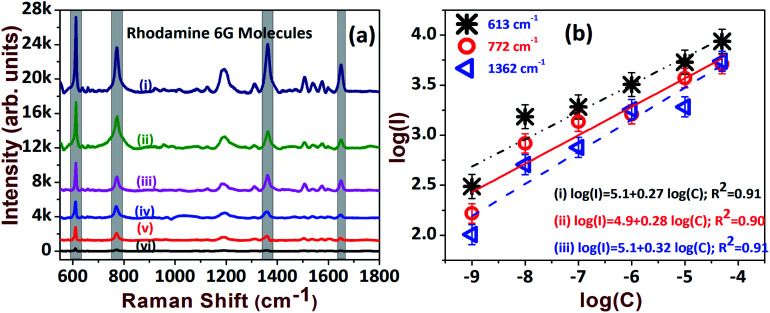
(a) The SERS spectra of R6G molecules tested at: (i) 50 μM; (ii) 10 μM; (iii) 1 μM; (iv) 0.1 μM; (v) 10 nM; (vi) 1 nM. (b) Linear dependence of the Raman intensity *versus* concentration for the prominent modes of R6G molecules. Spectra in (a) are displaced on the *Y*-axis for clarity.

The AgNDs prepared at a deposition temperature of 30 °C were utilized for further experimental investigations. Furthermore, to assess the sensing efficacy, the SERS substrates were tested with R6G probe molecules. The sensitivity of the substrate was examined at varying concentrations of the analyte molecule (50 μM to 1 nM) and the data are presented in Fig. 5a. We identified and were able to successfully assign the intense peaks of R6G observed at 613 cm^−1^, 772 cm^−1^ and 1362 cm^−1^. Fig. 5b shows the linear dependence of Raman scattering intensities as a function of concentration for the prominent peaks of R6G. It is evident that the SERS spectra show the prospective detection of an ultra-low concentration of 1 nM. Ten arbitrary spots were chosen to estimate the effective enhancement factor (EF) by adopting the conventional method of accounting for the adsorption factor *η*.^42,43^ The EF in the case of R6G with a concentration of 1 nM was estimated to be 8.5 × 10^7^ and was compared with existing reported values, as summarized in Table 1.

**Table 1:** Comparison of Ag and Ag/Au SERS substrates and their EFs

S. No.	SERS substrates	Analyte (concentration)	Peaks (cm^−1^)	EF	Ref.
1	Silver nanodendrites	R6G (10^−9^ M)	613	0.85 × 10^8^	This work
2	Silver nanocubes	R6G (10^−7^ M)	611	8.7 × 10^10^	45
3	Ultrafast laser photoreduction of Ag^+^	R6G (1 mM)	612	1 × 10^11^	46
4	Silver nanodendrites supported on Al sheets	R6G (10^−6^ M)	612	6.6 × 10^3^	47
5	Ag/pyramidal Si	R6G (10^−5^ M)	613	6.7 × 10^3^	48
6	Silver nanodendrites	RDX (5 × 10^−6^ M)	884	1.1 × 10^4^	This work
7	Silver nanocubes	RDX (10^−9^ M)	881	9.2 × 10^10^	45
8	Ag–Au alloy NPs loaded Si micro arrays	RDX (10^−6^ M)	883	9.3 × 10^4^	44
			1214	7.5 × 10^4^	
9	rGO–Ag nanocomposite	RDX (10^−12^ M)	880	1.0 × 10^9^	49
10	Gold nanoparticles	RDX (10^−5^ M)	882	1.0 × 10^4^	52
11	Silver nanodendrites	AN (10^−6^ M)	1048	3.1 × 10^4^	This work
12	Ag–Cu NPs	AN (5 mM)	1047	3.3 × 10^4^	50
13	AgNPs in PS nanofibers using electrospinning	RDX (10^−7^ M)	872	10^5^	51

**Fig. 6:**
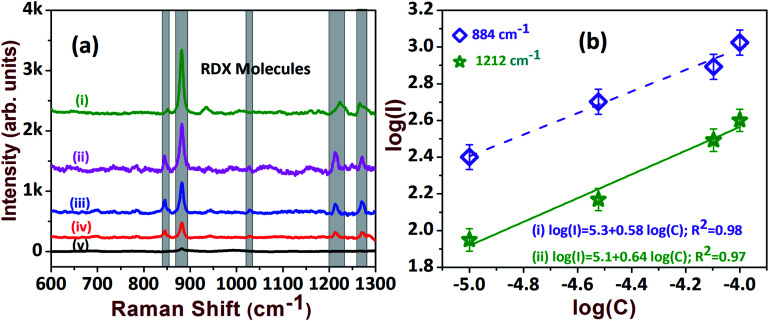
(a) The SERS spectra of RDX explosive at: (i) 100 μM; (ii) 80 μM; (iii) 30 μM; (iv) 10 μM; (v) 5 μM. (b) Corresponding linear relationship of intensity *vs.* concentration.

In view of safety concerns in the military and other areas, the detection of explosives at trace levels (μM or lower concentrations) is essential to avoid adversity. However, the detection of explosives such as RDX and AN is a tedious job, owing to matrix effects and the low sensitivity of current detection techniques. Therefore, we aimed to test the sensitivities of the fabricated SERS-active AgNDs substrates for detecting RDX and AN molecules. Fig. 6a presents the Raman spectra of RDX from 100 μM to 5 μM, illustrating the characteristic peaks of RDX at 884 cm^−1^, 1214 cm^−1^, and 1308 cm^−1^. The magnitude of these peaks was observed to increase as a function of analyte concentration. The intense peaks diminished while approaching the lower limit. The estimated EF for the lower detection concentration was ∼1.1 × 10^4^ as depicted in Table 1. The linear dependence was extracted from the log plot of intensity *versus* analyte concentration, which is presented in Fig. 6b. R6G dye is a fluorescent molecule and it has an absorption maximum near 530 nm. The strong Raman signal obtained for this molecule could be due to the resonance effect in the Raman signals, since excitation is at 532 nm. The common explosive molecules have lower Raman scattering cross-sections in comparison with dye molecules. Further, these molecules have no absorption at 532 nm. Hence, the dye molecules are expected to give more enhancement than explosives. Additionally, the orientation and vicinity of the analyte molecules on the nanostructure determines the enhancements.

**Fig. 7:**
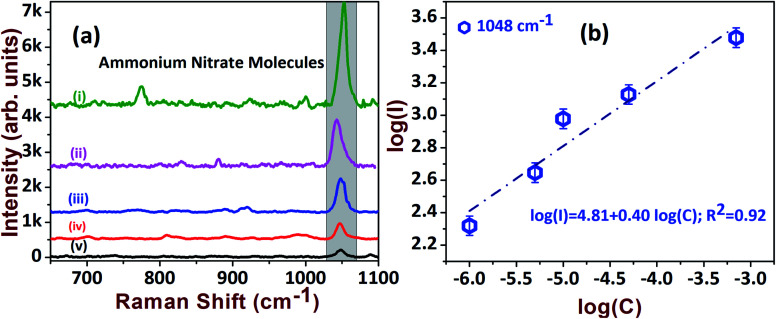
(a) The SERS spectra of AN explosive at: (i) 70 μM; (ii) 50 μM; (iii) 10 μM; (iv) 5 μM; (v) 1 μM. (b) Linear relationship of intensity *vs.* concentration.

The sensitive detection of AN molecules was also tested with the AgND substrate at various concentration levels. The intensity variations are clearly visualized by stacking the various concentrations of AN, which are presented in Fig. 7a. The trend between intensity and concentration followed a monotonic increment, which was extracted from a log plot of intensity *versus* concentration, as shown in Fig. 7b. The enhancement factor was estimated for 1 μM and found to be 3.1 × 10^4^ (see Table 1).

**Fig. 8:**
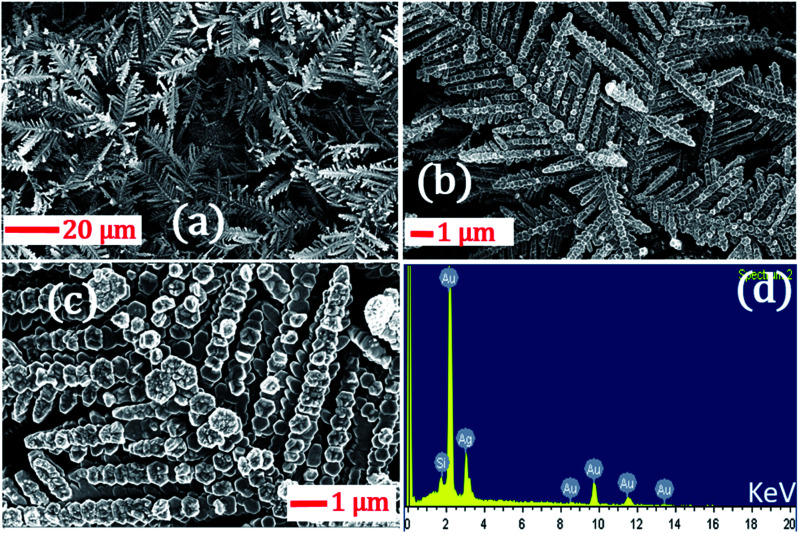
(a) FESEM images of the gold-deposited AgNDs at room temperature; (b) and (c) are higher magnification images of (a) for clarity. (d) EDX data of the AgNDs.

**Fig. 9:**
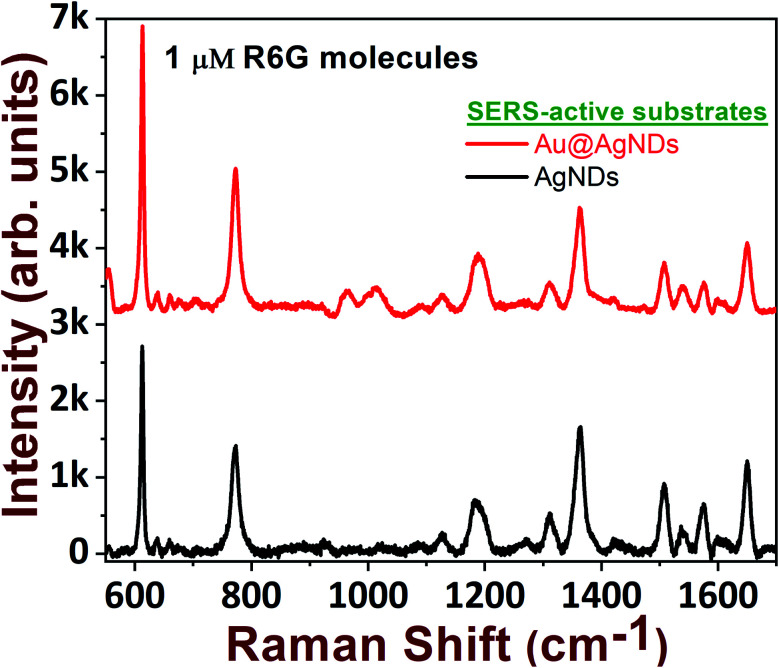
Black spectrum (bottom curve) represents the SERS spectrum of R6G molecules (1 μM concentration) recorded for pure AgNDs, while the red spectrum (top curve) represents the SERS spectrum obtained for Au@AgNDs.

In the foregoing discussion, it was demonstrated that the SERS-active AgNDs demonstrate excellent performance in the detection of dyes and explosives. However, these AgND substrates show a limitation in molecular detection at nM concentrations. Furthermore, silver nanostructures tend to oxidize over a long period of time, affecting the SERS signal and thereby limiting their application capability. To circumvent these difficulties, supplementary noble metal deposition was performed. The superior properties of gold and its compatibility with the rate of oxidation motivated us to track the gold deposition methodology on AgNDs for the prevention of prolonged oxidation and to enhance the stability of the substrates. The FESEM images are shown in Fig. 8. The gold deposition acts as a capping layer and can extensively enhance the sensing capabilities even at trace levels. Fig. 8b shows the higher magnification of Au-deposited AgNDs. The formation and arrangement of Au on the branches of AgNDs are visualized in Fig. 8c. The corresponding EDS spectrum is depicted in Fig. 8d. These SERS substrates were designed to be used for the detection of R6G at a concentration of 1 μM, as presented in Fig. 9. These results show that the synthesized Au-deposited AgNDs exhibit at least a two-fold enhancement for the 613 cm^−1^ Raman mode with an EF of 1.2 × 10^5^. These results highlight that the highly branched AgNDs and gold-deposited AgNDs can extensively be presented as cost-effective SERS-active substrates for trace-level molecular sensing.

**Fig. 10:**
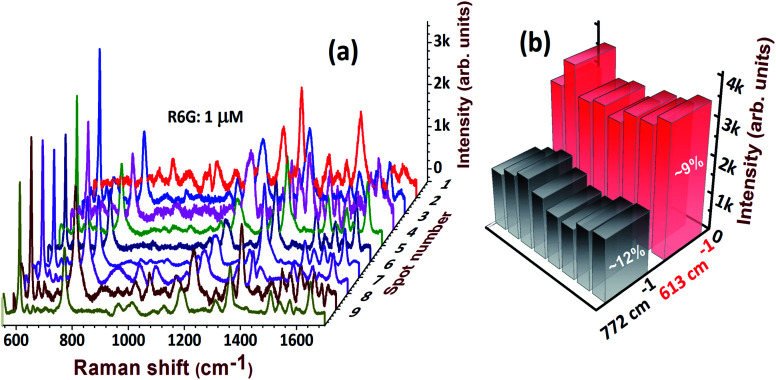
(a) Reproducibility of the SERS spectra of 1 μM R6G molecules detected at 10 different spots on AgNDs and (b) corresponding histogram with RSD values.

Reproducibility is an essential parameter to judge the quality and stability of SERS substrates. Fig. 10a illustrates the spectral reproducibility of the most dominant and detectable peaks of R6G: at 613 cm^−1^ and 772 cm^−1^. The corresponding histogram at 10 spots of detection is shown in Fig. 10b and relative standard deviation (RSD) values were estimated to be ∼9%, and ∼12%, respectively. In the commercial realization of SERS substrates, the foremost considerations are the following parameters: (a) reproducibility; (b) versatility; (c) cost-effectiveness; (d) stability; and (e) recyclability, *etc.* In the present case, we have successfully demonstrated the qualities (a)–(c). We believe that our substrates are stable for 4 weeks in normal storage conditions and could further be improved by coating with Au NPs or a compatible protective layer. We performed the Raman measurements on freshly-prepared and two-month-old substrates. We observed that 80% of the Raman signal was retained for the two-month-old substrates. Moreover, the EDX data on these substrates demonstrated that only ∼3.5% Ag was formed on the surface after two months of exposure to ambient conditions. The recyclability is possible by a simple NaBH_4_ chemical dipping. This is because NaBH_4_ has a strong ability to clean the stains caused by analyte molecules. The recyclability of AgND and Au@AgND substrates will be extensively investigated in our future works.

**Fig. 11:**
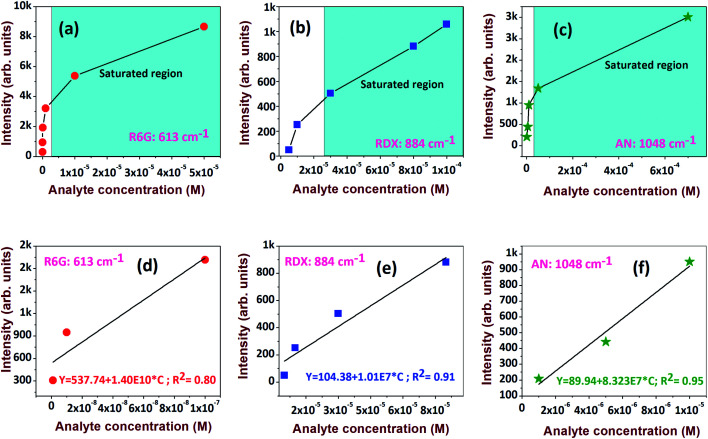
Raman intensity *versus* concentration for (a) R6G, 613 cm^−1^; (b) RDX, 884 cm^−1^; (c) AN, 1048 cm^−1^. (d–f) Linear dependence of the SERS intensities of corresponding molecules at lower analyte concentrations.

The investigation was progressed further to estimate the limit of detection (LOD) for the analytes. The prominent peaks at 613 cm^−1^, 884 cm^−1^, and 1048 cm^−1^ for R6G, RDX, and AN, respectively, were considered for exploration. The Raman intensity *versus* analyte concentration data and its linear fit are presented in Fig. 11a–c and d–f, respectively. The LOD^44^ is expressed as 3*σ*/*b*, where *σ* is the standard deviation of a non-SERS substrate and *b* is the slope derived from the linear plot at lower concentrations (Fig. 11d–f). The extracted LODs were ∼0.48 nM for R6G, ∼2 μM for RDX, and ∼350 nM for AN.

The sensitivity and capabilities of these substrates can be further enriched by tuning the EF and, subsequently, the LOD. Table 1 summarizes some of the recent SERS substrates based on Ag along with their composition and the obtained EFs for different analyte molecules.^44–52^ We demonstrated that the designed AgNDs and Au-decorated AgNDs exhibit superior performance in the field of molecular detection. The extensive detection of other explosive molecules in addition to the size and density effects of AuNPs^53,54^ on these substrates will be accomplished in forthcoming investigations. Furthermore, we will also study measures for improving the EFs using SERS efficient Ag–Au NPs, as demonstrated in our earlier works.^44^ Another challenge we will attempt to overcome is to detect these hazardous materials in mixtures^44,55^ or embedded in a matrix.

## 4. Conclusions

In summary, we have fabricated highly branched silver nanodendrites with different densities by a simple electroless deposition process. The density of AgNDs could be controlled by tuning the AgNO_3_ concentration. Morphological changes were also observed as a function of AgNO_3_ deposition temperature. The optimized SERS-active AgNDs enhance the Raman spectrum of Rhodamine 6G (R6G) with a reasonably large enhancement factor, *i.e.*, ∼10^8^. The capabilities of the substrates were further analyzed by performing the detection of the explosives RDX and AN. The calculated EFs for R6G (1 nM), RDX (5 μM), and AN (1 μM) were ∼10^8^, ∼10^4^, and ∼10^4^, respectively. Furthermore, through simple Au deposition on AgNDs, we demonstrated a noteworthy improvement in the sensitivity of detection for R6G. We firmly believe that the sensitivity of detection can be further enriched by varying the size and density of the AuNPs on AgNDs.

## 5. Data availability statement

The data that support the findings of this study are available from the corresponding author upon reasonable request.

## Conflicts of interest

There are no conflicts to declare.

## Supplementary Material
